# The Evaluation of the Phytochemical Profiles and Antioxidant and α-Glucosidase Inhibitory Activities of Four Herbal Teas Originating from China: A Comparative Analysis of Aqueous and Ethanol Infusions

**DOI:** 10.3390/foods13111705

**Published:** 2024-05-29

**Authors:** Jin Zhang, Jinling Lv, Guodong Zhuang, Junjia Zhang, Feng Hu, Yongsheng Chen

**Affiliations:** 1 Department of Food Science and Engineering, Jinan University, Guangzhou 510632, Chinaljlhhh0412@163.com (J.L.);; 2 Dr. Neher’s Biophysics Laboratory for Innovative Drug Discovery, State Key Laboratory of Quality Research in Chinese Medicine, Macau University of Science and Technology, Macao; 3 Department of Food Science, Rutgers, The State University of New Jersey, 65 Dudley Road, New Brunswick, NJ 08901, USA

**Keywords:** herbal tea, phytochemicals, antioxidant, α-glucosidase inhibition

## Abstract

Recent research has demonstrated the positive impact of herbal tea consumption on postprandial blood glucose regulation. This study conducts a comparative analysis of aqueous and ethanol extractions on four herbal teas (Mallotus, Cyclocarya, Rubus, and Vine) to assess their phytochemical profiles and functional attributes. Phytochemical contents, antioxidant activities, α-glucosidase inhibitory activities, and chemical compositions are investigated via colorimetric analyses and UPLC-Q-Orbitrap HRMS/MS, respectively. Results indicate that Vine, among the teas studied, exhibits the most pronounced glucose-regulating effects under both extraction methods. While ethanol extractions yield higher phytochemical content overall, the compositions vary. Conversely, aqueous extracts demonstrate unexpectedly potent antioxidant activities and comparable α-glucosidase inhibitory activities to ethanol extracts. Phytochemical contents correlate positively with antioxidant activities and α-glucosidase inhibitory activities. However, antioxidant activities exhibit a weak positive correlation with α-glucosidase inhibitory activities. These findings provide evidence that aqueous extracts from herbal teas contain valuable phytochemical compositions beneficial for antioxidants and individuals with hyperglycemia, suggesting their potential as functional ingredients to enhance the nutritional value of herbal food products.

## 1. Introduction

Diabetes mellitus is a type of metabolic disorder with hyperglycemia that can induce disruptions of insulin secretion [1]. Meanwhile, postprandial hyperglycemia is a symptom of diabetes mellitus, and the regulation of postprandial hyperglycemia indeed plays a vital role in the treatment of diabetic patients. For instance, studies have found that regardless of whether an individual is diabetic or unaffected, α-glucosidase plays a crucial role in the adjustment of postprandial hyperglycemia [2]. With increasing research interests regarding α-glucosidase, the presence of α-glucosidase has been found within the epithelium of the human intestines, and the inhibition of such α-glucosidase could serve as potential targets of postprandial hyperglycemia regulations for diabetic patients. As a result, the inhibition of α-glucosidase enzyme activities has become a promising strategy to regulate blood glucose levels [3] and, therefore, has triggered great research interest in natural α-glucosidase inhibitors for the well-being of diabetic patients.

In recent years, there has been growing interest, both among consumers and researchers, in the health benefits associated with the consumption of plant-based products. This interest is partly fueled by the abundance of health-promoting active ingredients found in plants and plant-based products, such as dietary phytochemicals, known for their numerous beneficial effects [4]. Additionally, numerous epidemiological and preclinical studies have highlighted the potential of herbal tea consumption in reducing the risk of chronic diseases including cancer, cardiovascular disease [5], diabetes mellitus [6], and more. China stands out as a major producer of tea, offering a wide variety of high-quality teas globally. Traditionally, tea has been derived from the leaves and/or leaf buds of *Camellia sinensis*. However, herbal teas made from leaves, flowers, fruits, roots, and other components of various herbs or plants have been prepared for infusion worldwide, such as with the male papaya flower [7], *Eucommia ulmoides* [8], and magnolia flower. Herbal teas with antioxidative and antidiabetic properties are consumed for their health-promoting benefits across different regions, exhibiting significant industrial value [9]. Importantly, herbal tea consumption and its associated benefits have been advocated as alternatives to pharmacological interventions for improving health and alleviating symptoms of certain diseases [10]. Consequently, there is a growing demand for research aimed at elucidating the physiological activities of herbal tea and its phytochemical constituents, with potential applications spanning pharmaceuticals, agriculture, and the food industry. Previous studies have commonly employed aqueous and organic solvent extractions to isolate phytochemical compounds [11,12], enabling the extraction of a diverse array of phytochemicals from plants [13]. Ethanol extracts of phytochemicals have been noted for their potent bioactivities [14,15], although aqueous extraction is more prevalent in daily consumption and has demonstrated health effects on the human body.

In general, phenolics have the capacity to interact with metabolic enzymes in the human body, potentially influencing their activities [16]. For example, research has unveiled the impact of interactions between dietary phenolics and metabolic enzymes on human health [17], emphasizing the importance of exploring the mechanisms underlying such interactions. While there have been studies investigating the interactions between flavonoids and α-glucosidase, research on the interactions between phytochemical constituents from non-camellia herbal teas and α-glucosidase remains scarce. Therefore, further investigations into these interactions are essential to elucidate their health benefits and practical applications. Mallotus, Cyclocarya, Rubus, and Vine (Table 1) are popular herbal tea choices in China and several Southeast Asian countries due to their flavor profiles and perceived beneficial effects on the human body, including antioxidant and antidiabetic activities [18,19]. However, the hypoglycemic activity of these four herbal teas extracted using different extraction methods remains relatively unclear. In particular, exploring the correlation between antioxidant and hypoglycemic effects is essential for gaining a deeper understanding of the health-promoting functionalities of herbal teas.

In light of the above reasoning, this study was aimed to evaluate the total phenolics, total flavonoids, and total flavonol content of extracts, as well as the respective antioxidant activities and inhibitions of α-glucosidase activity of the four herbal tea extracts extracted with hot-water and ethanol methods (Table 1). Moreover, the phytochemical profiles of the four herbal tea extracts were further investigated through in-depth HPLC-DAD-Q-Orbitrap HRMS/MS analyses.

## 2. Materials and Methods

### 2.1. Chemicals

Gallic acid was obtained from Sigma-Aldrich (Saint Louis, MO, USA). Rutin was bought from Shanghai Macklin Biochemical Co., Ltd., (Shanghai, China). Catechin and 2,2′-azinobis(3-ethyl-benzothiazoline-6-sulfonic acid) diammonium salt (ABTS) were acquired from Aladdin Biochemical Technology Co., Ltd., (Shanghai, China). 1,1-Diphenyl-2-picrylhydrazyl (DPPH) was obtained from Shanghai Yuanye Biotechnology Co., Ltd., (Shanghai, China). The other reagents were analytically pure.

### 2.2. Sample Preparation

Four herbal teas were bought from the local market (Table 1). Specifically, Mallotus tea (*Mallotus oblongifolius* (*Miq.*) *Müll. Arg.*) was yielded from Baoting, Hainan Province, China; Rubus tea (*Rubus savissimus S. Lee*) was planted in Jinxiu, Guangxi Province, China; Cyclocarya tea (*Cyclocarya paliurus*) was produced in Xiangxi, Hunan Province, China; and Vine tea (*Ampelopsis grossedentata*) was obtained from Zhangjiajie, Hunan Province, China. All herbal teas were crushed at room temperature and passed through a screen with a 60-mesh sieve; the powders were stored at −25 °C until further analysis.

The aqueous extraction proceeded as follows: the herbal tea powder (1 g, 60-mesh) was blended with 50 mL of boiling distilled water in a 95 °C water bath for 6 min before vacuum filtration, and the residue was repeated twice. All collected prefiltration fluid was transferred to a volumetric flask (200 mL) with distilled water to the 200 mL tick mark and stored at −20 °C until analysis. The organic extraction proceeded as follows: the herbal tea powder (1 g, 60-mesh) was blended with 50 mL of ethyl alcohol at 60 °C for 30 min before vacuum filtration, and the residue was repeated twice. All collected prefiltration fluid was transferred to a volumetric flask (200 mL) with distilled water to the 200 mL tick mark and stored at −20 °C until analysis.

### 2.3. Determination of Total Phenolic Content (TPC)

The TPC was determined using the Folin–Ciocalteu reagent method following a previous report [20]. Briefly, tested extracts or gallic acid solution were mixed with Folin–Ciocalteu reagent and kept for 6 min at ambient temperature; then, they were blended with Na_2_CO_3_ (7%) solution and purified water, respectively. After 90 min at ambient temperature, the final mixture would be measured at 760 nm. With gallic acid employed as a standard, the total phenolic content was shown as milligrams of gallic acid equivalent (GAE) per gram of dry weight (DW).

### 2.4. Determination of Total Flavonoid Content

The total flavonoid content was analyzed through NaNO_2_-AlCl_3_ colorimetric assay according to a previous report [21]. Briefly, samples and standard solution were mixed with NaNO_2_ (5%, *w*/*v*) and purified water, respectively. Kept for 5 min at ambient temperature, AlCl_3_ (10%, *w*/*v*) working solution was blended with the mixture. After another 6 min, NaOH (1 M) solution and purified water were added to a colorimetric tube, respectively, and the resulting mixture was read at 510 nm. With catechin as the reference, the total flavonoid content was shown as milligrams of catechin equivalent (CE) per gram of DW.

### 2.5. Determination of Total Flavonol Content

The total flavonol content was measured using a NaOAc-AlCl_3_ colorimetric method [22]. Briefly, extracts and standard solution were blended with AlCl_3_ working solution (20 mg/mL) and sodium acetate solution (50 mg/mL), respectively. Kept for 150 min, the absorbance of the mixture was immediately measured at 440 nm. Rutin was employed as the standard, and the flavonol content was expressed as mg rutin equivalent (RE)/g DW.

### 2.6. Determination of 2,2-Diphenyl-1-picrylhydrazyl Radical (DPPH) Scavenging

The DPPH scavenging assay was carried out according to a previous report [23]. Briefly, extracts and standard solution were mixed with DPPH working solution (1:1, *v*/*v*) and kept in the dark for 30 min. The absorbance was analyzed at 515 nm. Results were compared against the standard curve prepared with quercetin and were expressed as mg quercetin equivalents (QE)/g DW.

### 2.7. Determination of 2,2′-Azino-bis(3-ethylbezothiazoline-6-sulfonic acid) Radical Cation (ABTS^•+^) Scavenging

The ABTS assay was performed in referring to a previous study [24]. The radical cation working solution was prepared through ABTS [2,2′-azinobis-(3-ethylbenzothiazoline-6-sulfonic acid) diammonium salt] (7 mM) and K_2_S_2_O_8_ (140 mM). The absorbance of ABTS working solution was diluted to 0.700 ± 0.020 using phosphate (50 mM). Extracts and standard solution were blended with the ABTS working solution in the dark for 10 min, and the resulting mixtures’ absorbance was analyzed at 734 nm. Inhibition values were obtained according to the following equation:

$${\mathrm{Inhibition}\;\mathrm{of}\;A}_{734}\;\left(\%\right)=\left(1-A_{t}/A_{0}\right)\times 100$$

where A_t_ = absorbance of the samples and A_0_ = absorbance of the control. Results were calculated using a standard curve of Trolox and expressed as mmol TE/g DW.

### 2.8. Determination of α-Glucosidase Inhibition In Vitro

The analysis of α-glucosidase inhibition was performed with the previously described method [2]. In brief, α-glucosidase was prepared with phosphate-buffered saline (0.1 M, pH 6.8), and 4-nitrophenyl-α-D-glucopyranoside (*p*-NPG) was applied as the substrate. Pre-diluted tea infusion was mixed with α-glucosidase solution for 10 min at 37 °C. The reaction was started with the addition of the substrate, and the mixture was maintained for 20 min under 37 °C. In the end, 1 mL of absolute ethanol was added into the mixture as an enzyme inhibitor for p-nitrophenyl release, and the absorbance was measured at 405 nm. Blank and positive controls were established with absolute ethanol and acarbose, respectively. The rate of inhibition (%) toward α-glucosidase was calculated as below:

$$\mathrm{Inhibition}\left(\%\right)=\frac{A_{c}-A_{s}}{A_{c}}\times 100$$

where A_c_ and A_s_ represent the absorbance of the control and sample, respectively. Substrate was present in all these groups. Inhibitory activity is expressed as efficient concentration EC_50_: the sample concentration (μg/mL) required to obtain 50% activity.

### 2.9. Determination of Enzyme Kinetics Assays for α-Glucosidase

The reversible assays of herbal tea extracts against α-glucosidase were established through a concentration gradient of *p*-NPG with varying concentrations of herbal tea extract, respectively. For the determination of the specific inhibition kinetic, the velocity of the reaction was carried out at different concentrations of substrate and herbal tea extract. In the mode of α-glucosidase inhibition assay, a gradient of 0, 0.2, 0.4, 0.6, 0.8, and 1 mM *p*-NPG was applied as the substrate. Lineweaver–Burk plots were employed to determine the inhibition kinetics, including the plot of 1/ν versus 1/[S].

### 2.10. UPLC-Q-Orbitrap HRMS/MS Analysis

UPLC analysis was performed with the Dionex 3000 Ultimate UPLC system equipped with an auto-sampler and was coupled with a Q-Exactive Orbitrap HRMS/MS (Thermo Fisher, Waltham, MA, USA). Acquity UPLC BEH C18 column (2.1 mm × 100 mm, 1.8 μm) was applied and operated at 35 °C. Mobile phase composition followed (A) 0.1% formic acid in water and (B) acetonitrile with a flow rate of 0.3 mL/min.

MS analysis was carried out with a heated electrospray ionization source under negative ionization mode. The mass parameters were established as follows: auxiliary gas flow, 10 arb; sheath gas flow, 45 arb; spray voltage, 3500 V; mass scan range, *m*/*z* 100–1000; auxiliary gas heater temperature, 100 °C; capillary temperature, 350 °C. A data-dependent program was picked for tandem mass spectrometry data acquisition, and the most affluent precursor ions were chosen for MS/MS analysis. The collision energy of collision-induced dissociation and high-collision energy dissociation mode was limited to 35% of the maximum.

#### 2.10.1. Cyclocarya Tea

The elution parameters were 0–5 min, 5–18% B; 5–8 min, 18–20% B; 8–13 min, 20–25% B; 13–18 min, 25–80% B; and 18–20 min, 80–80% B, followed by 5 min of re-equilibration.

#### 2.10.2. Mallotus Tea

The elution parameters were 0–7 min, 5–10% B; 7–21 min, 10–20% B; and 21–24 min, 20–50% B, followed by 5 min of re-equilibration.

#### 2.10.3. Rubs Tea

The elution parameters were: 0–6 min, 5–10% B; 6–20 min, 10–15% B; 20–30 min, 15–20% B; and 30–34 min, 20–50% B, followed by 5 min of re-equilibration.

#### 2.10.4. Vine Tea

The elution parameters were: 0–3 min, 5–12% B; 3–11 min, 12–15% B; 11–16 min, 15–30% B; and 16–20 min, 30–80% B, followed by 5 min of re-equilibration.

### 2.11. Statistical Analysis

Statistical analyses were performed using SPSS 26.0 software (SPSS Inc., Chicago, IL, USA). The significance was fixed at *p* < 0.05. All data were presented as the means ± SD from at least three replications.

## 3. Results and Discussion

### 3.1. Phytochemicals in Herbal Extract

#### 3.1.1. Total Phenolic Content

**Figure 1:**
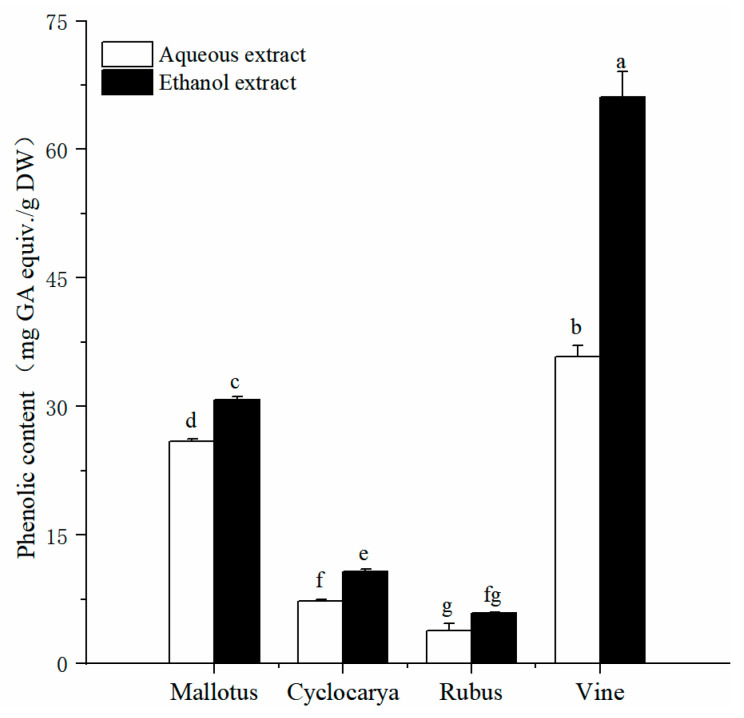
Total phenolic contents of four herbal tea varieties (means ± SD, *n* = 3). Bars with different letters differ significantly at *p* < 0.05.

Exploration of the phytochemical compositions of herbal teas would pvovide useful information for their potential application, while phenolic is one of the common phytochemicals within herbal teas. Phenolics have garnered increasing attention from consumers and researchers due to their demonstrated health effects [25]. Figure 1 illustrates the detected total phenolic content in aqueous and ethanol extracts. The results reveal significant differences in the content of active ingredients among the four herbal tea extracts obtained via both extraction methods (*p* < 0.05). Total phenolic content values range from 3.86 ± 0.79 to 66.12 ± 2.98 mg GAE/g DW (Figure 1), with Vine exhibiting the highest phenolic content. Specifically, aqueous extraction yields the highest TPC value for Vine (35.77 ± 1.31 mg GAE/g DW, *p* < 0.05), followed by Mallotus > Cyclocarya > Rubus. Similarly, ethanol extraction shows Vine with the highest TPC value (66.12 ± 2.98 mg GAE/g DW), followed by Mallotus > Cyclocarya > Rubus. Significant differences in total phenolic content are observed among the four herbal tea varieties (*p* < 0.05). The total phenolic contents obtained from the two extracts are comparable to those of Nettle (*Urtica dioica*) leaves using methanol, water, and ethanol extractions [26]. However, the total phenolic contents of Mallotus and Vine are lower than those of the young leaves of *M. toringoides* [27], which could be attributed to differences in testing methods. Additionally, the total phenolic contents of Rubus leaves are lower than those of the Rubus fruits, which are approximately one-tenth [28]. Factors such as origin, variety, harvest time, parts used, extraction method, and analytical technique may contribute to variations in phenolic content among the tested samples. Overall, the phenolic content assay results demonstrate that ethanol extraction yields higher phenolic content, suggesting that ethanol extraction may be preferred for extracting phenolics from herbal teas.

#### 3.1.2. Total Flavonoid Content

**Figure 2:**
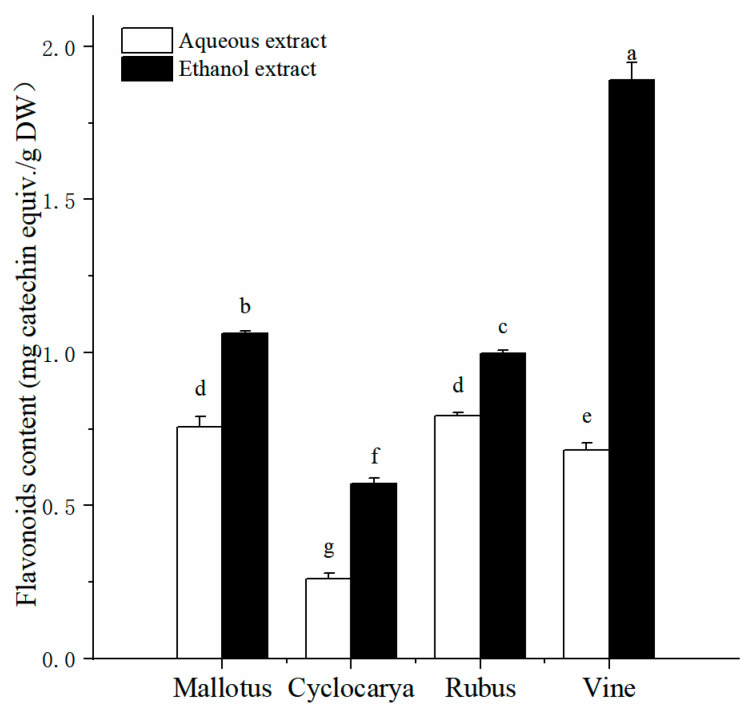
Total flavonoid contents of four herbal tea varieties (means ± SD, *n* = 3). Bars with different letters differ significantly at *p* < 0.05.

Flavonoids are ubiquitous dietary phytochemicals found in plant-based foods, including teas [20], fruits [29], and vegetables [30]. Previous epidemiological studies have indicated that a higher intake of flavonoids may reduce the risk of chronic diseases [31]. Figure 2 presents the total flavonoid content values obtained from the herbal tea extracts, with notably higher values obtained using the ethanol extraction method. Across different herbal tea varieties, the flavonoid content ranges from 0.26 ± 0.02 mg CE/g DW to 1.89 ± 0.06 mg CE/g DW. Interestingly, the pattern of flavonoid content among the four herbal tea extracts differs from that of the phenolic content. Vine, extracted using ethanol, exhibits the highest total flavonoid content (1.89 ± 0.06 mg CE/g DW, *p* < 0.05) among all samples, followed by Mallotus > Rubus > Cyclocarya. Conversely, Rubus shows the highest total flavonoid content (0.79 ± 0.01 mg CE/g DW, *p* < 0.05) from aqueous extraction, followed by Mallotus > Vine > Cyclocarya. Most of the tested samples have higher total flavonoid contents compared to Lemon Balm (*Melissa officinalis* L.) herbal tea in hot and/or cold extracts [32]. However, the total flavonoid contents of most tested samples are similar to those of highly consumed leaf teas and herbal infusions in Spain [33], suggesting minimal variation in leaf samples from different origins. Once again, these results affirm that ethanol extraction is a preferred method for extracting flavonoids from herbal teas.

#### 3.1.3. Total Flavonol Content

**Figure 3:**
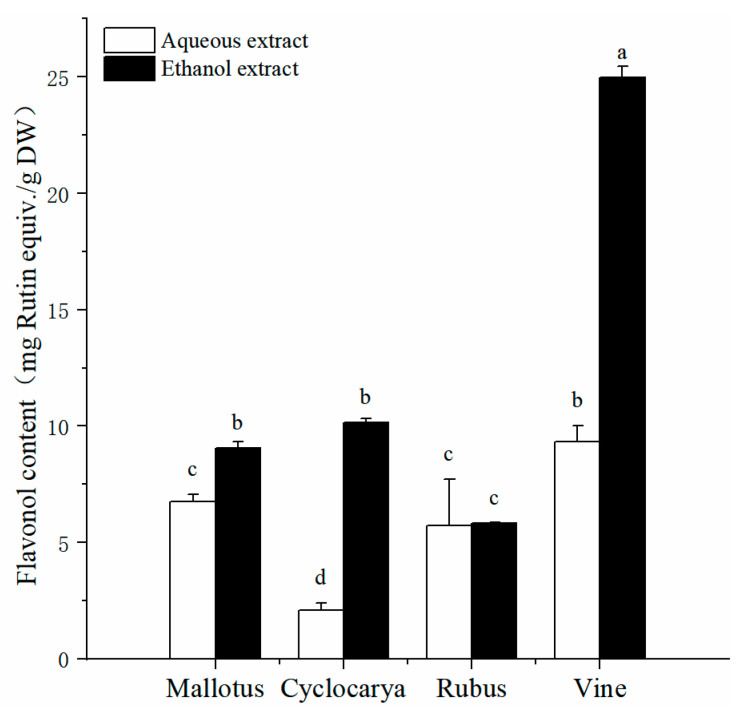
Total flavonol contents of four herbal tea varieties (means ± SD, *n* = 3). Bars with different letters differ significantly at *p* < 0.05.

Flavonols, a subgroup of flavonoids [34], are widely distributed in plant-derived products such as teas, grapes, onions, and others. *In vitro* studies have confirmed the potent antioxidant and anti-inflammatory activities of flavonols [35]. Figure 3 presents the determined total flavonol content from four herbal tea extracts obtained using ethanol and aqueous methods. Total flavonol content values varied among the different herbal tea extracts, ranging from 2.09 ± 0.33 RE/g DW to 24.98 ± 0.49 mg RE/g DW. Ethanol extraction resulted in Vine displaying the highest total flavonol content (24.98 ± 0.50 mg RE/g DW, *p* < 0.05), while Cyclocarya from aqueous extraction exhibited the lowest total flavonol content (2.09 ± 0.33 RE/g DW, *p* < 0.05). Specifically, with ethanol extraction, Vine showed the highest total flavonol content (*p* < 0.05), followed by Cyclocarya > Mallotus > Rubus. Similarly, with aqueous extraction, Vine also exhibited the highest total flavonol content, followed by Mallotus > Rubus > Cyclocarya. The recommended daily intake of flavonol for adults is approximately 29 mg [36], and results from the total flavonol content analysis suggest that Vine tea could serve as one of the best dietary sources of flavonol among the four herbal teas. Moreover, the flavonol contents from most ethanol extracts were comparable to those of Camellia tea leaves [37], highlighting the value of ethanol extraction as a method for obtaining dietary flavonols.

### 3.2. Percentage Contribution of Flavonoid/Flavonol to Phenolics

**Figure 4:**
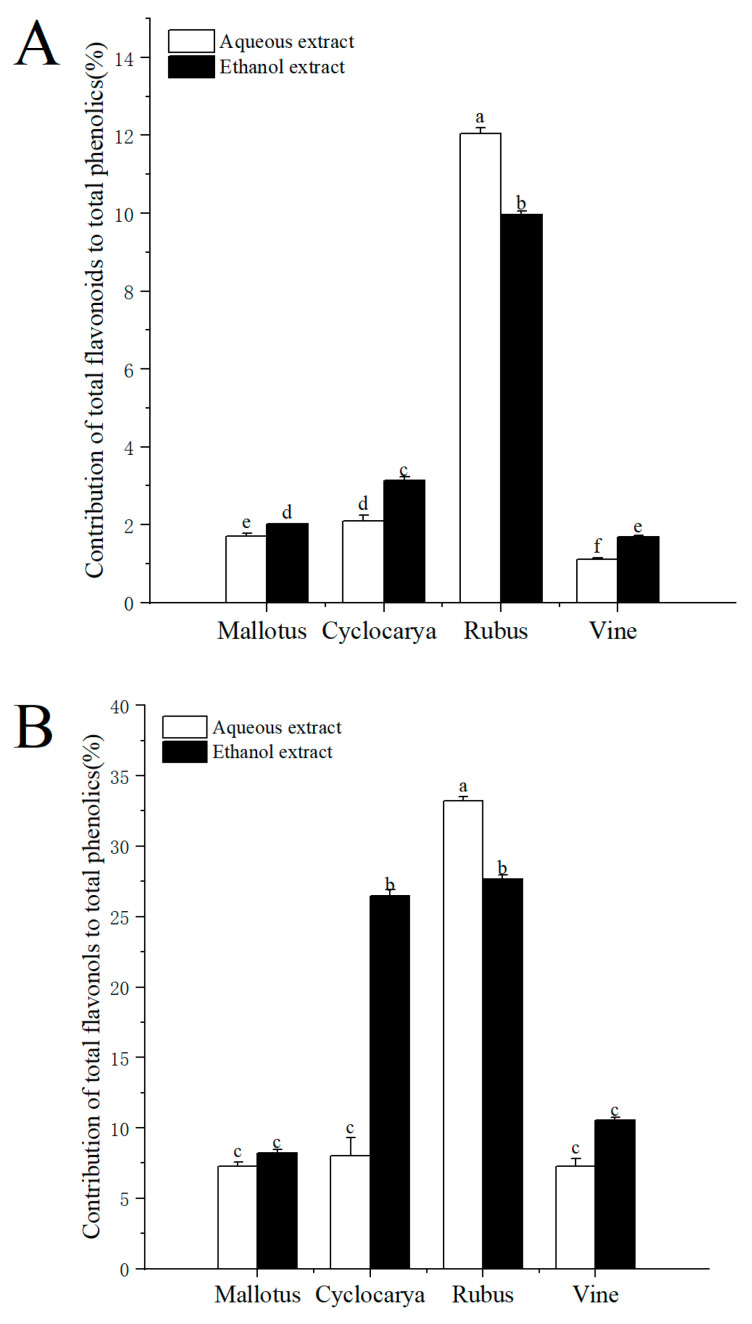
Percent contribution of flavonoids to phenolics (**A**), percent contribution of flavonols to phenolics (**B**) (means ± SD, *n* = 3). Bars with different letters differ significantly at *p* < 0.05.

On a molar basis, the percentage contribution of flavonoid/flavonol to phenolics was estimated and demonstrated in Figure 4. The contributions of total flavonoids to total phenolics ranged from 1.12 to 12.05%, indicating flavonoids composed of only a small part of phenolics for the four herbal teas. At the same time, the contribution of flavonoids to phenolics was the highest in the aqueous extract of Rubus (12.05 ± 0.14%), followed by the ethanol extract of Rubus (9.96 ± 0.10%), then ethanol extract of Cyclocarya (3.13 ± 0.10%), and was lowest in the aqueous extract of Vine (1.12 ± 0.04%). However, the percentage contribution of flavonoids to phenolics of the four tested herbal teas was lower than that of Adinandra leaves [20]. The factors affecting the phenolic and flavonoid levels within herbal teas could be season, location, climate, variety, species, the age of the leaf plucked, manufacturing conditions and processes, and particle sizes [38]. Gao et al. [39] also pointed out that flavonoids tend to be unstable when exposed to high temperatures and light for long periods of time, which could result in inactivation and/or degradation. However, tea processes almost always involve light and heat, which may cause different degrees of flavonoid loss in tea and lead to varied testing results. Besides flavonoids, the contributions of total flavonols to total phenolics ranged from 7.25 to 33.20%, indicating that flavonols made up a larger part of phenolics in the four herbal teas. The contribution of flavonols to phenolics was the highest in the aqueous extract of Rubus (33.20 ± 0.30%) and the lowest in the aqueous extract of Mallotus (7.25 ± 0.34%). Nevertheless, there were no significant differences among the three samples: Cyclocarya ≈ Vine ≈ Mallotus, indicating flavonols were one of the major phenolics within Euphorbiaceae. With ethanol extract, the contribution of flavonols to phenolics was the highest in Rubus (27.67 ± 0.30%), followed by Cyclocarya > Vine ≈ Mallotus. The percentage contribution of flavonols to phenolics was higher than that of flavonoids to phenolics, which showed that the four tested herbal teas were good potential sources of dietary flavonol, especially Rubus aqueous extract. Overall, the results demonstrated that ethanol extract was beneficial for obtaining phytochemicals from the herbal teas.

### 3.3. Antioxidant Activity

**Figure 5:**
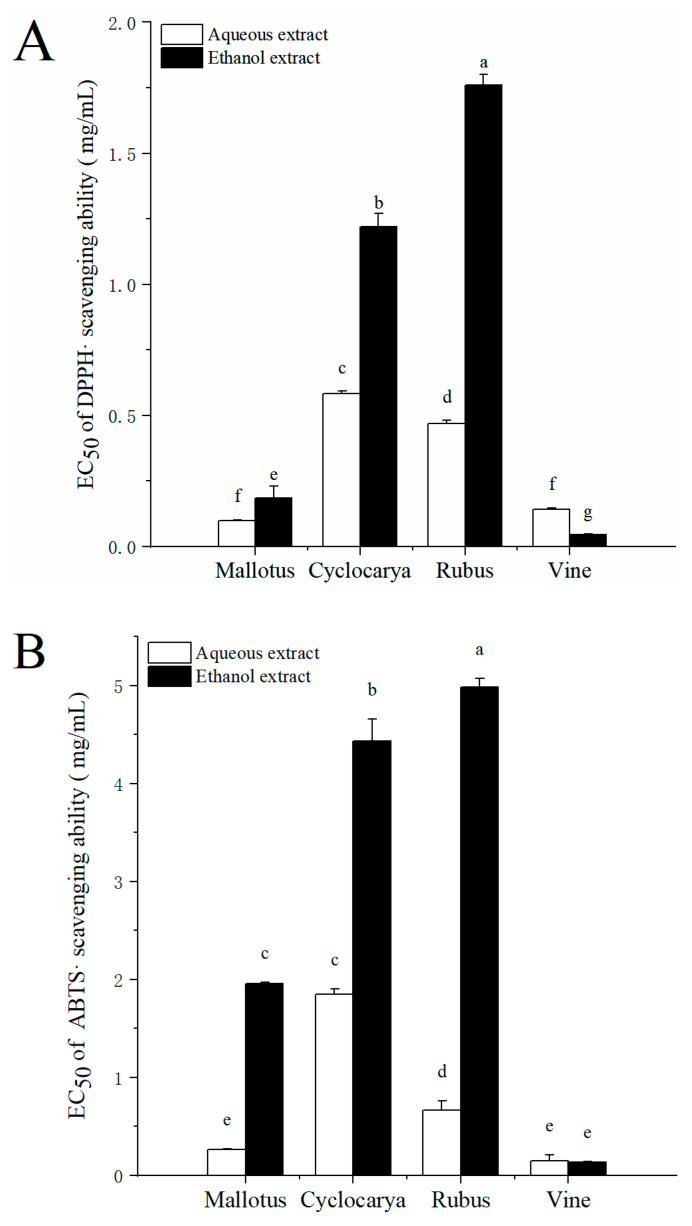
DPPH (**A**) and ABTS (**B**) EC_50_ values of four herbal tea varieties (means ± SD, *n* = 3). Bars with different letters differ significantly at *p* < 0.05.

Research has demonstrated that supplemental antioxidants can mitigate the overproduction of free radicals and play critical roles in promoting good health [40]. Therefore, investigating the antioxidant activities of natural plant products is important for the development of plant-based functional foods [41]. Phytochemicals present in herbal teas are known for their potent antioxidant properties, among other beneficial effects on human health [42]. *In vitro* antioxidant activities were assessed using the DPPH radical scavenging assay and ABTS^•+^ radical cation scavenging assay. The DPPH radical scavenging abilities of different extracts are presented as EC_50_ value in Figure 5A. Generally, a lower EC_50_ value indicates a higher radical scavenging ability. Among all samples, the ethanol extract of Vine exhibited the lowest EC_50_ value (0.045 ± 0.002 mg/mL, *p* < 0.05), while the ethanol extract of Rubus had the highest EC_50_ value (1.76 ± 0.04 mg/mL, *p* < 0.05), indicating the highest and lowest DPPH radical scavenging abilities in Vine and Rubus with ethanol extraction, respectively. The trend of the DPPH radical scavenging ability with ethanol extraction was followed by Rubus < Cyclocarya < Mallotus < Vine. Conversely, through aqueous extraction, the DPPH radical scavenging ability followed Cyclocarya < Rubus < Vine < Mallotus. Interestingly, the DPPH EC_50_ values of the four tested herbal teas were lower than those of the green and black teas originating from Indonesia [43], indicating a stronger DPPH radical scavenging activity in the four herbal teas. As shown in Figure 5B, the ABTS^•+^ radical cation scavenging activity mirrored that of the DPPH radical scavenging ability: in ethanol extracts, the ABTS^•+^ radical cation scavenging activity was ranked as Rubus < Cyclocarya < Mallotus < Vine, while in aqueous extracts, it was ranked as Cyclocarya < Rubus < Mallotus < Vine. Interestingly, most aqueous extracts displayed stronger antioxidant activity than ethanol extracts. These results suggest that aqueous extracts may offer greater antioxidant benefits for human health, contrary to the previous analysis results regarding phytochemical contents. Thus, there may be interactions among the bioactive substances present in herbal teas extracted using aqueous methods.

### 3.4. α-Glucosidase Inhibitory Activity

**Figure 6:**
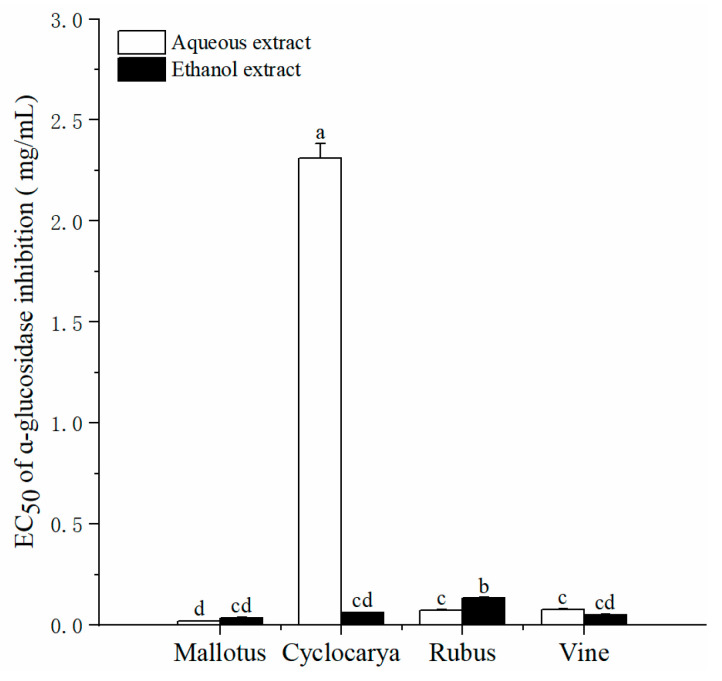
α-Glucosidase inhibitory activity EC_50_ values of four herbal tea varieties (means ± SD, *n* = 3). Bars with different letters differ significantly at *p* < 0.05.

α-Glucosidase is among the effective carbohydrate-hydrolyzing enzymes, with α-glucosidase inhibitors capable of regulating postprandial carbohydrates hydrolysis and lowering postprandial glucose levels. Studies have indicated that α-glucosidase inhibitors derived from diet can be advantageous for long-term glycemic control [44]. As depicted in Figure 6, the EC_50_ values of α-glucosidase inhibition varied significantly among the four herbal tea extracts, ranging from 0.02 ± 0.001 to 2.31 ± 0.07 mg/mL. Except for the aqueous extract of Cyclocarya, other herbal tea extracts demonstrated potent inhibitory activities. The inhibitory effect of aqueous extracts on α-glucosidase activity was ranked as Mallotus > Rubus ≈ Vine > Cyclocarya, while that of ethanol extracts was ranked as Mallotus ≈ Vine ≈ Cyclocarya > Rubus. Similar to antioxidant activity, most ethanol extracts exhibited comparable α-glucosidase inhibition activity to aqueous extracts, with no significant difference. These findings suggest that the components inhibiting α-glucosidase are similar to those exhibiting antioxidant properties. Previous studies have indicated that although phytochemicals are readily extracted with ethanol solvents [45], high photochemical contents may not necessarily result in high antioxidant activity. Since herbal tea is typically steeped in hot water for daily consumption and phytochemicals are extracted into aqueous solvents, these results imply that aqueous extraction methods of herbal tea can indeed provide active substances for health benefits.

### 3.5. Inhibitory Types

**Figure 7:**
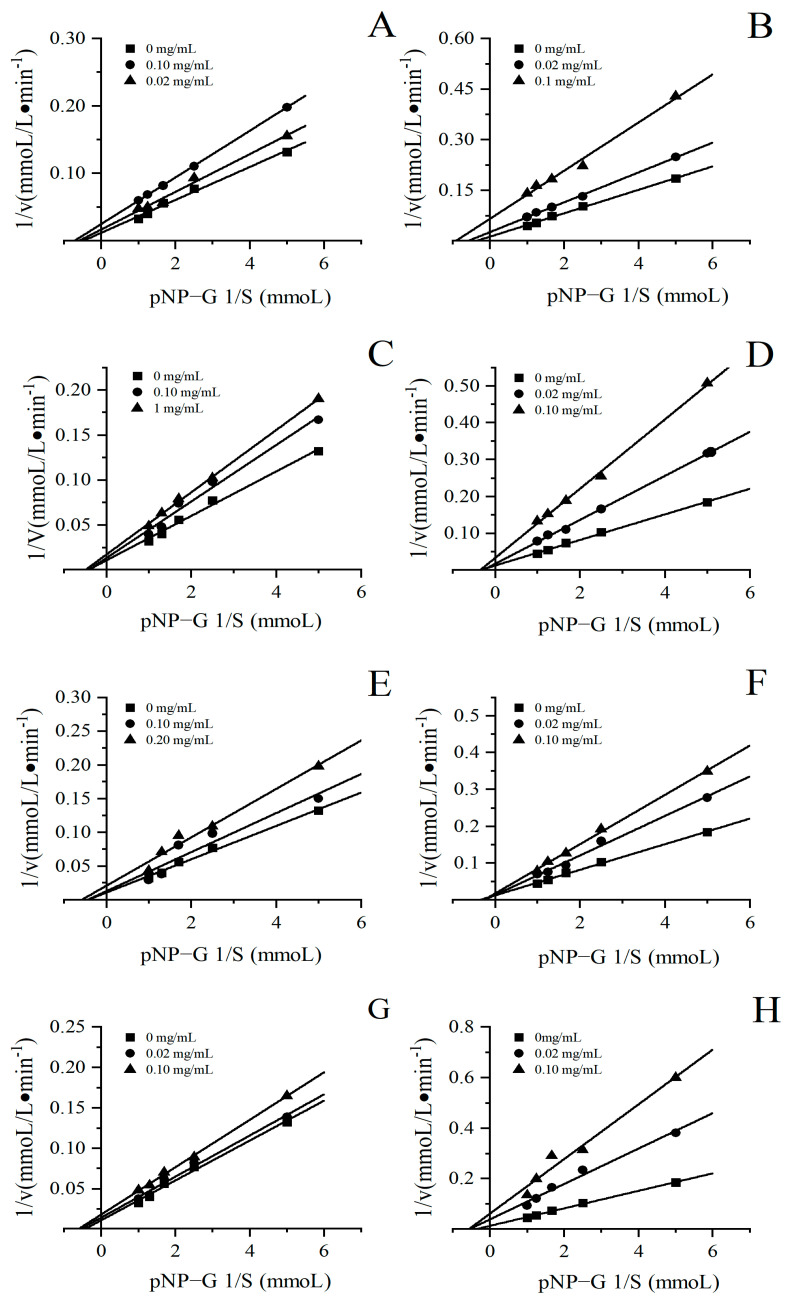
Lineweaver–Burk plots of four herbal tea variety extracts for determining the inhibitory mode. (**A**,**C**,**E**,**G**) are the double reciprocal curves of the aqueous extracts of partridge, Cyclocarya paliurus, sweet tea and vine tea, respectively; (**B**,**D**,**F**,**H**) are the double reciprocal curves of the ethanolic extracts of partridge, Cyclocarya paliurus, sweet tea and vine tea, respectively.

To further analyze the inhibition types of the four herbal tea extracts on α-glucosidase, the sample concentration and substrate concentration were first fixed, and the enzymatic reaction was carried out in the same reaction system. According to the Lineweaver–Burk and Dixon double reciprocal curve mapping method, the double reciprocal curve was drawn to determine the type of enzymatic reaction competition. The double reciprocal curves are displayed in Figure 7. The point of intersection of the straight line with the y-axis represents 1/V_max_, and the point of intersection of the straight line with the x-axis represents 1/K_m_. The slope of this line is K_m_/V_max_K. As shown in Figure 7, the enzymatic reaction rate V changes with the substrate concentration [S]. For instance, during the enzymatic reaction, with the increase in sample concentration, the apparent Michaelis constant K_m_ remained unchanged and the initial velocity V_m_ of the maximum catalytic reaction decreased, indicating that the inhibition type of the four herbal tea extracts on α-glucosidase was non-competitive reversible inhibition. The inhibition types of the four herbal teas differ from Nigerian green tea leaves [46], which indicated that herbal teas and green tea had different bioactive constituents on α-glucosidase inhibition.

### 3.6. Correlation between Phytochemical and Activities

Given that the stronger the activity of the four herbal tea extracts, the smaller the EC_50_ value, 1/EC_50_ is utilized to represent the activities of the samples when analyzing the correlation between the active ingredients and the antioxidant and hypoglycemic activities. Considering that thousands of phytochemicals contribute to biological activities [6,47], it remains unclear which substance is primarily responsible for the tested activity. Table 2 presents the results of the Pearson correlation coefficient between the active components of herbal teas and their antioxidant and hypoglycemic activities. The total phenolics content showed significant and positive correlations with DPPH radical scavenging and ABTS^•+^ radical scavenging abilities (*p* < 0.01), consistent with previous findings [47]. The correlation coefficients were 0.903 ** and 0.756 **, respectively, indicating that tea phenolics were key factors influencing the antioxidant activity of the herbal teas. Moreover, total phenolic content exhibited a positive correlation with the inhibition of α-glucosidase, suggesting that the *in vitro* hypoglycemic activity was linked to the total polyphenol contents, consistent with previous reports [6]. Additionally, the DPPH free radical scavenging effect showed a significant and positive correlation with the ABTS value (0.750, *p* < 0.01), suggesting mutual corroboration between these two methods of evaluating antioxidant activity. Both the DPPH free radical scavenging ability and ABTS scavenging ability also displayed positive correlations with α-glucosidase inhibition. The consistent use of herbal plant antioxidants to enhance endogenous antioxidant self-defense mechanisms could be a promising approach to alleviate oxidative stress in diabetic patients by reducing free radicals, and increasing the intake of herbal plant antioxidants could potentially boost the activity of endogenous antioxidant enzymes [48,49]. However, it is puzzling that the total flavonoid and total flavonol contents exhibited negative correlations with the ABTS scavenging ability and α-glucosidase inhibition. These correlation results suggest that flavonoids and flavonols may inhibit the ABTS scavenging ability and α-glucosidase inhibition. Conversely, the correlation indicates that the α-glucosidase inhibition and antioxidant activity of the herbal teas appear to be significantly influenced by polyphenols, aligning with previous reports [6,47].

### 3.7. UPLC-Q-Orbitrap HRMS/MS Analysis

**Figure 8:**
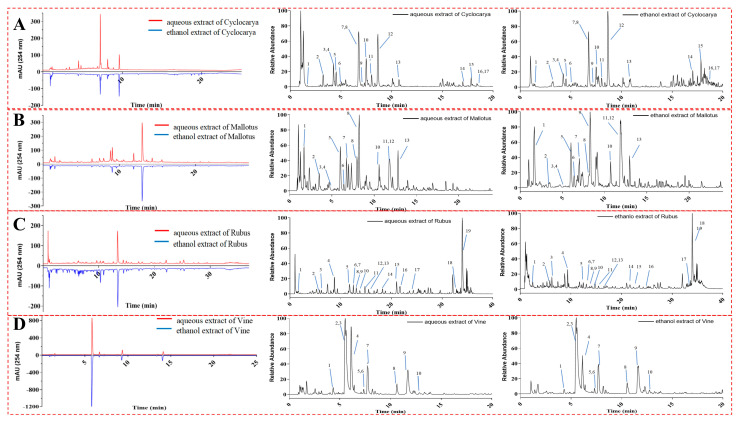
HPLC-DAD chromatogram of four herbal tea varieties extracts. ((**A**): Cyclocarya, 1–17 listed in Table 3 and Table 4; (**B**): Mallotus, 1–14 lisetd in Table 5 and Table 6; (**C**): Rubus, 1–19 listed in Table 7 and Table 8; (**D**): Vine, 1–10 listed in Table 9 and Table 10).

In addition to previous investigations, the rapid UPLC-Q-Orbitrap HRMS/MS analytical coupling technique was further employed to offer additional insights into the chemical compositions of phytochemicals extracted using water and/or ethanol from the four herbal teas. Specifically, UPLC-Q-Orbitrap HRMS/MS provides comprehensive insights into the chemical structures and compositions of complex natural compounds with high specificity and sensitivity. The chromatogram results, presented in Figure 8, thoroughly analyzed the precursor and product ions of compounds found in the four herbal teas, along with their corresponding mass and composition. For instance, representative compounds in the four herbal teas were tentatively identified by comparing the analyzed retention behavior, HRMS/MS data, and mass fragment characteristics with respective reference compounds from previous studies and/or available works in the literature. Characterizations of these compounds are detailed in Table 3, Table 4, Table 5, Table 6, Table 7, Table 8, Table 9 and Table 10, suggesting distinct chemical constituents among the extracts from the four herbal teas. Seventeen phytochemicals were identified from Cyclocarya aqueous and ethanol extracts, with most classified as glycosides, indicating that glycosides were the main bioactive compounds, consistent with previous studies [50]. Similarly, for Mallotus, thirteen and fourteen phytochemicals were identified from the aqueous and ethanol extracts, respectively, with glycosides also predominating, consistent with prior studies [51,52]. Rubus yielded nineteen and eighteen identified phytochemicals from the aqueous and ethanol extracts, respectively. These results could explain Rubus’s higher percentage contribution of flavonoids to phenolics, stronger antioxidant activities, and more potent α-glucosidase inhibition. Previous data showed that Vine had the highest total phenolic constituents among the four herbal teas, along with the strongest activity. However, only ten compounds were identified in Vine. It is plausible that synergistic effects within Vine’s chemical constituents, which could enhance biological activity [41,42], might be present. Dihydromyricetin, identified from Vine, has been shown to contribute to α-glucosidase inhibition [53], suggesting potential synergistic effects among dihydromyricetin and other substances in Vine.

## 4. Conclusions

The current study aimed to comparatively evaluate aqueous and ethanol extracts from four selected herbal teas. Ethanol extracts exhibited higher contents of phenolics, flavonoids, and flavonols in terms of phytochemicals. The contributions of flavonoids and flavonols to phenolics from ethanol extractions were greater than those from aqueous extractions. Interestingly, aqueous extraction enhanced the antioxidant activity of most herbal tea samples and demonstrated similar α-glucosidase inhibitory activity to ethanol extraction. Correlation results indicated a positive association between polyphenols and antioxidant and α-glucosidase inhibitory activities. Generally, aqueous extraction should be considered an efficient and cost-effective processing method to enhance the bioactivities of the four herbal teas in the natural functional food and natural medicine industries. Specifically, results suggested that Vine had the highest phytochemical contents, antioxidant activity, and α-glucosidase inhibitory activity across both aqueous and ethanol extractions. This study underscores the importance of aqueous extractions in herbal tea consumption. Additionally, further research will focus on exploring the structure–activity relationship and synergistic effects within the aqueous extracts.

## Figures and Tables

**Table 1:** Descriptions of the four herbal teas employed in the study.

Figure of Teas	Common Name	Chinese Name	Species	Family	Part Used and Packaging
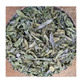	Mallotus	Shankucha	*Mallotus peltatus*	Euphorbiaceae	Leaf, Loose tea
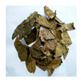	Cyclocarya	Qingqianliu	*Cyclocarya paliurus*	Juglandaceae	Leaf, Loose tea
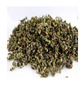	Rubus	Tiancha	*Rubus chingii*	Rosaceae	Leaf, Loose tea
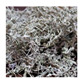	Vine	Meicha	*Ampelopsis grossedentata*	Vitaceae	Leaf, Loose tea

**Table 2:** Correlation analysis of 1/EC_50_ value and various indices of tea extracts from different varieties.

Index	Total Polyphenols	Total Flavonoids	Total Flavonols	DPPH Scavenging Ability	ABTS Scavenging Ability	α-Glucosidase Inhibition
Total Polyphenols	1.00	0.05	0.04	0.903 **	0.756 **	0.32
Total Flavonoids		1.00	0.872 **	0.06	−0.15	−0.12
Total Flavonols			1.00	0.02	−0.13	−0.19
DPPH Scavenging Ability				1.00	0.750 **	0.33
ABTS Scavenging Ability					1.00	0.17
α-Glucosidase Inhibition						1.00

** Very significant correlation at the *p* < 0.01 level.

**Table 3:** Chemical constituents in aqueous extract of Cyclocarya.

No.	Compound	RT	Expected	Measured	Adduct Ion	Formula	Delta	MS2 Ion
		(min)	(*m*/*z*)	(*m*/*z*)			(ppm)	(*m*/*z*)
1	Gallic acid	1.68	153.0184	170.0215	[M-H]^−^	C_7_H_6_O_5_	1.077	124.0155, 79.0177
2	Neochlorogenic acid	3.25	353.0882	354.0951	[M-H]^−^	C_16_H_18_O_9_	4.224	191.0557, 179.0344, 135.0442
3	4-caffeoylquinic acid	4.26	353.0883	354.0951	[M-H]^−^	C_16_H_18_O_9_	4.649	191.0557, 179.0344, 135.0442
4	Catechin	4.30	289.0723	290.0790	[M-H]^−^	C_15_H_14_O_6_	5.761	NA
5	5-caffeoylquinic acid	4.53	353.0882	354.0951	[M-H]^−^	C_16_H_18_O_9_	4.309	191.0557, 161.0233
6	Epicatechin	5.23	289.0723	290.0790	[M-H]^−^	C_15_H_14_O_6_	5.554	NA
7	Hyperin	6.68	463.0888	464.0955	[M-H]^−^	C_21_H_20_O_12_	3.752	301.0338, 271.0252
8	Quercetin-3-O-glucuronide	6.74	477.0678	478.0747	[M-H]^−^	C_21_H_18_O_13_	3.088	301.0347, 151.0028
9	Isoquercitrin	6.81	463.0888	464.0955	[M-H]^−^	C_21_H_20_O_12_	3.687	301.0338, 271.0252
10	Kaempferol-3-O-glucuronide	7.48	461.0732	462.0798	[M-H]^−^	C_21_H_18_O_12_	3.747	285.0409, 229.0508, 113.0231
11	4,5-O-dicaffeoylquinic acid	8.03	515.1201	516.1268	[M-H]^−^	C_25_H_24_O_12_	3.218	353.0883, 179.0344, 173.0449
12	Afzelin	8.65	431.0988	432.1056	[M-H]^−^	C_21_H_20_O_10_	3.519	285,0402, 255.0301, 227.0348
13	Quercetin	10.88	301.0361	302.0427	[M-H]^−^	C_15_H_10_O_7_	4.521	121.0029, 107.0127
14	Cyclocaric acid B	17.48	485.3279	486.3345	[M-H]^−^	C_30_H_46_O_5_	3.501	NA
15	Cyclocarioside I	17.84	621.4016	622.4080	[M-H]^−^	C_35_H_58_O_9_	3.058	621.4023, 471.3488
16	Cyclocarioside III	18.27	635.4177	636.4237	[M-H]^−^	C_36_H_60_O_9_	3.714	635.4179, 489.3565
17	Cyclocaroside II	19.37	603.3919	604.3975	[M-H]^−^	C_35_H_56_O_8_	4.582	NA

NA: not assessed.

**Table 4:** Chemical constituents in ethanol extract of Cyclocarya.

No.	Compound	RT	Expected	Measured	Adduct Ion	Formula	Delta	MS2 Ion
		(min)	(*m*/*z*)	(*m*/*z*)			(ppm)	(*m*/*z*)
1	Gallic acid	1.68	169.0135	170.0215	[M-H]^−^	C_7_H_6_O_5_	0.380	125.0234, 97.0283, 79.0176
2	Neochlorogenic acid	3.22	353.0883	354.0951	[M-H]^−^	C_16_H_18_O_9_	4.479	191.0556, 179.0344, 135.0442
3	4-caffeoylquinic acid	4.25	353.0882	354.0951	[M-H]^−^	C_16_H_18_O_9_	4.139	191.0556, 179.0344, 135.0442
4	Catechin	4.25	289.0722	290.0790	[M-H]^−^	C_15_H_14_O_6_	5.208	NA
5	5-caffeoylquinic acid	4.52	353.0882	354.0951	[M-H]^−^	C_16_H_18_O_9_	4.309	191.0557, 179.0344, 135.0442
6	Epicatechin	5.23	289.0723	290.0790	[M-H]^−^	C_15_H_14_O_6_	5.208	NA
7	Hyperin	6.68	463.0889	464.0955	[M-H]^−^	C_21_H_20_O_12_	3947	301.0339, 271.0252
8	Quercetin-3-O-glucuronide	6.74	477.0679	478.0747	[M-H]^−^	C_21_H_18_O_13_	3.151	301.0347
9	Isoquercitrin	6.81	463.0890	464.0955	[M-H]^−^	C_21_H_20_O_12_	4.141	301.0339, 271.0252
10	Kaempferol-3-O-glucuronide	7.48	461.0732	462.0798	[M-H]^−^	C_21_H_18_O_12_	3.812	285.0409, 229.0508, 113.0231
11	4,5-O-dicaffeoylquinic acid	8.02	515.1201	516.1268	[M-H]^−^	C_25_H_24_O_12_	3.334	353.0883, 179.0344, 173.0449
12	Afzelin	8.66	431.0987	432.1056	[M-H]^−^	C_21_H_20_O_10_	3.287	285,0402, 255.0301, 227.0348
13	Quercetin	10.86	301.037	302.0427	[M-H]^−^	C_15_H_9_O_7_	4.853	121.0029, 107.0127
14	Cyclocaric acid B	17.48	485.3276	486.3345	[M-H]^−^	C_30_H_46_O_5_	3.068	NA
15	Cyclocarioside I	17.84	621.4015	622.4080	[M-H]^−^	C_35_H_58_O_9_	2.865	471.3485
16	Cyclocarioside III	18.27	635.4169	636.4237	[M-H]^−^	C_35_H_60_O_9_	2.377	489.3584
17	Cyclocaroside II	19.38	603.3911	604.3975	[M-H]^−^	C_35_H_56_O_8_	3.257	NA

NA: not assessed.

**Table 5:** Chemical constituents in aqueous extract of Mallotus.

No.	Compound	RT	Expected	Measured	Adduct Ion	Formula	Delta	MS2 Ion
		(min)	(*m*/*z*)	(*m*/*z*)			(ppm)	(*m*/*z*)
1	Gallic acid	1.71	169.0134	170.0215	[M-H]^−^	C_7_H_6_O_5_	1.303	125.0233, 97.0283, 79.0176
2	Caffeic acid 3-beta-d-glucuronide	3.49	355.0673	356.0743	[M-H]^−^	C_15_H_16_O_10_	3.878	209.0305, 191.2879, 85.0282
3	Catechin	5.27	289.0721	290.0790	[M-H]^−^	C_15_H_14_O_6_	4.793	NA
4	Epicatechin	5.39	289.0721	290.0790	[M-H]^−^	C_15_H_14_O_6_	5.104	NA
5	Phyllanthusiin A	6.00	291.0149	292.0219	[M-H]^−^	C_13_H_8_O_8_	4.523	247.0246
6	Caffeic acid	6.41	179.0343	180.0422	[M-H]^−^	C_9_H_8_O_4_	2.093	135.0444.
7	Repandusinic acid A	6.73	969.0856	970.0924	[M-H]^−^	C_41_H_30_O_28_	1.696	633.0741, 463.0522, 247.0249
8	Geraniin	7.97	951.0747	952.0818	[M-H]^−^	C_41_H_28_O_27_	1.288	933.0649, 463.0527, 445.0410
9	Corilagin	8.24	633.0737	634.0806	[M-H]^−^	C_27_H_22_O_18_	2.259	463.0520, 169.0138
10	Phyllanthusiin C	10.58	925.0957	926.1025	[M-H]^−^	C_40_H_30_O_26_	1.668	605.0793, 453.0683, 247.0253, 169.0133
11	Ellagic acid	11.78	300.9990	302.0063	[M-H]^−^	C_14_H_6_O_8_	3.742	257.0089, 245.0085, 201.0188
12	Kaempferol-3-O-sophoroside	11.98	593.1517	594.1585	[M-H]^−^	C_27_H_30_O_15_	2.754	NA
13	Rutin	12.83	609.1464	610.1534	[M-H]^−^	C_27_H_30_O_16_	2.214	NA

NA: not assessed.

**Table 6:** Chemical constituents in ethanol extract of Mallotus.

No.	Compound	Rt	Expected	Measured	Adduct Ion	Formula	Delta	MS2 Ion
		(min)	(*m*/*z*)	(*m*/*z*)			(ppm)	(*m*/*z*)
1	Gallic acid	1.68	169.0133	170.0215	[M-H]^−^	C_7_H_6_O_5_	1.126	125.0234, 97.0283, 79.0176
2	Cyanidin	2.34	371.0622	372.0693	[M-H]^−^	C_15_H_16_O_11_	3.429	
3	Caffeic acid 3-beta-d-glucuronide	3.47	355.0674	356.0743	[M-H]^−^	C_15_H_16_O_10_	3.962	209.0298, 191.0194, 85.0282
4	Catechin	5.21	289.0721	290.0790	[M-H]^−^	C_15_H_14_O_6_	5.000	NA
5	Epicatechin	5.27	289.0721	290.0790	[M-H]^−^	C_15_H_14_O_6_	4.793	NA
6	Phyllanthusiin E	6.00	291.0147	292.0219	[M-H]^−^	C_13_H_8_O_8_	3.905	247.0246
7	Caffeic acid	6.38	179.0342	180.0422	[M-H]^−^	C_9_H_8_O_4_	1.926	135.0443.
8	Repandusinic acid A	6.80	969.0858	970.0924	[M-H]^−^	C_41_H_30_O_28_	1.820	633.0747, 463.0519, 247.0246
9	Geraniin	7.97	951.0746	952.0818	[M-H]^−^	C_41_H_28_O_27_	1.218	933.0633, 463.0529, 445.0432
10	Corilagin	8.28	633.0736	634.0806	[M-H]^−^	C_27_H_22_O_18_	2.164	463.0523, 169.0132
11	Phyllanthusiin C	10.58	925.0957	926.1025	[M-H]^−^	C_40_H_30_O_26_	1.668	605.0793, 453.0699, 247.0247, 169.0135
12	Ellagic acid	11.86	300.9991	302.0063	[M-H]^−^	C_14_H_6_O_8_	4.041	257.0091, 245.0091, 201.0189
13	Kaempferol-3-O-sophoroside	12.08	593.1516	594.1585	[M-H]^−^	C_27_H_30_O_15_	2.451	NA
14	Rutin	12.49	609.1469	610.1534	[M-H]^−^	C_27_H_30_O_16_	3.117	NA

NA: not assessed.

**Table 7:** Chemical constituents in aqueous extract of Rubus.

No.	Compound	RT	Expected	Measured	Adduct Ion	Formula	Delta	MS2 Ion
		(min)	(*m*/*z*)	(*m*/*z*)			(ppm)	(*m*/*z*)
1	Gallic acid	1.67	169.0134	170.0215	[M-H]^−^	C_7_H_6_O_5_	1.717	NA
2	Brevifolincarboxylic acid	5.83	291.0149	292.0219	[M-H]^−^	C_13_H_8_O_8_	4.317	247.0246, 191.0343, 173.0236
3	Caffeic acid	6.19	179.0343	180.0423	[M-H]^−^	C_9_H_8_O_4_	2.149	117.0331
4	Ferulic acid hexoside	8.76	355.1036	356.1107	[M-H]^−^	C_16_H_20_O_9_	3.609	161.0235, 133.0285
5	Ellagic acid	11.74	300.9991	302.0063	[M-H]^−^	C_14_H_6_O_8_	4.174	NA
6	Rutin	12.51	609.0898	610.1534	[M-H]^−^	C_27_H_30_O_16_	2.822	301.0349, 300.0280, 271.0258
7	Quercetin	12.83	301.0357	302.0427	[M-H]^−^	C_15_H_10_O_7_	4.654	NA
8	Quercetin-O-hexoside	13.04	463.0888	464.0955	[M-H]^−^	C_21_H_20_O_12_	3.623	271.0248, 151.0028
9	Isoquercitrin	13.07	463.0887	464.0955	[M-H]^−^	C_21_H_20_O_12_	3.472	NA
10	Quercitrin	13.78	463.0889	464.0955	[M-H]^−^	C_21_H_20_O_12_	3.947	NA
11	Kaempferol-3-O-rutinoside	14.78	593.1517	594.1585	[M-H]^−^	C_27_H_30_O_15_	2.754	285.0395, 257.0467, 151.0030
12	Quercetin-3-O-α-d-ribofuranoside	15.51	433.0780	434.0849	[M-H]^−^	C_20_H_18_O_11_	3.377	NA
13	Kaempferol-O-hexoside	15.56	447.0940	448.1006	[M-H]^−^	C_21_H_20_O_11_	3.964	284.0329, 255.0200, 227.0348, 151.0025
14	Kaempferol-O-pentoside	18.27	417.0831	417.0822	[M-H]^−^	C_20_H_17_O_10_	3.469	284.0329, 255.0300, 227.0347,
15	Caffeic acid-O-dihexoside	21.02	503.1202	504.1268	[M-H]^−^	C_24_H_24_O_12_	3.642	341.0881, 281.0671, 251.0560, 221.0454, 179.0344, 161.0236, 135.0442
16	Quercetin-O-caffeyl-hexoside	21.74	625.1207	626.1272	[M-H]^−^	C_30_H_26_O_15_	2.997	463.0888, 301.0356, 161.0237
17	Kaempferol-O-caffeoyl-hexoside	24.33	609.1258	610.1323	[M-H]^−^	C_30_H_26_O_14_	3.133	447.0947, 323.0778, 285.0408, 161.0237
18	Kaempferol	32.76	285.0409	334.0325	[M-H]^−^	C_15_H_10_O_9_	5.352	285.0409, 151.0023
19	Rubusoside	33.87	641.3178	642.3251	[M-H]^−^	C_32_H_50_O_13_	1.625	479.2658, 317.2126

NA: not assessed.

**Table 8:** Chemical constituents in ethanol extract of Rubus.

No.	Compound	RT	Expected	Measured	Adduct Ion	Formula	Delta	MS2 Ion
		(min)	(*m*/*z*)	(*m*/*z*)			(ppm)	(*m*/*z*)
1	Gallic acid	1.66	169.0134	170.0215	[M-H]^−^	C_7_H_6_O_5_	1.658	125.0233,
2	Brevifolincarboxylic acid	5.83	291.01498	292.0219	[M-H]^−^	C_13_H_8_O_8_	4.317	247.0246, 191.0343, 173.0236
3	Caffeic acid	6.24	179.0342	180.0423	[M-H]^−^	C_9_H_7_O_4_	1.647	135.0442, 117.0334
4	Ferulic acid hexoside	8.76	355.1036	356.1107	[M-H]^−^	C_16_H_20_O_9_	3.440	161.0235, 133.0285
5	Ellagic acid	11.74	300.9991	302.0063	[M-H]^−^	C_14_H_6_O_8_	3.842	NA
6	Rutin	12.44	609.1465	610.1534	[M-H]^−^	C_27_H_30_O_16_	2.411	301.0349, 300.0280, 271.0258
7	Quercetin	12.83	301.0357	302.0427	[M-H]^−^	C_15_H_10_O_7_	4.654	NA
8	Quercetin-O-hexoside	13.06	463.0886	464.0955	[M-H]^−^	C_21_H_20_O_12_	3.148	271.0248, 151.0028
9	Isoquercitrin	13.07	463.0887	464.0955	[M-H]^−^	C_21_H_19_O_12_	3.472	NA
10	Quercitrin	13.78	463.0889	464.0955	[M-H]^−^	C_21_H_20_O_12_	3.947	NA
11	Kaempferol-3-O-rutinoside	14.74	593.1516	594.1585	[M-H]^−^	C_27_H_30_O_15_	2.451	285.0395, 257.0467, 151.0030
12	Quercetin-3-O-α-D-ribofuranoside	15.51	433.0778	434.0849	[M-H]^−^	C_20_H_18_O_11_	2.961	NA
13	Kaempferol-O-hexoside	15.56	447.0940	448.1006	[M-H]^−^	C_21_H_20_O_11_	3.964	284.0329, 255.0200, 227.0348, 151.0025
14	Caffeic acid-O-dihexoside	21.01	503.1200	504.1268	[M-H]^−^	C_24_H_24_O_12_	3.235	341.0881, 281.0671, 251.0560, 221.0454, 179.0344, 161.0236, 135.0442
15	Quercetin-O-caffeyl-hexoside	21.67	625.1205	626.1272	[M-H]^−^	C_30_H_25_O_15_	2.789	463.0888, 301.0356, 161.0237
16	Kaempferol-O-caffeoyl-hexoside	24.27	609.1255	610.1323	[M-H]^−^	C_30_H_25_O_14_	2.739	447.0947, 323.0778, 285.0408, 161.0237
17	Kaempferol	32.74	285.0410	334.0325	[M-H]^−^	C_15_H_10_O_9_	5.562	285.0409, 151.0023
18	Rubusoside	33.95	641.3186	642.3251	[M-H]^−^	C_32_H_50_O_13_	2.857	479.2658, 317.2126

NA: not assessed.

**Table 9:** Chemical constituents in aqueous extract of Vine.

No.	Compound	RT	Expected	Measured	Adduct Ion	Formula	Delta	MS2 Ion
		(min)	(*m*/*z*)	(*m*/*z*)			(ppm)	(*m*/*z*)
1	Catechin	4.38	289.0721	290.0790	[M-H]^−^	C_15_H_14_O_6_	4.896	203.0712, 125.0223
2	Dihydromyricetin	5.53	319.0461	320.0532	[M-H]^−^	C_15_H_12_O_8_	3.875	257.0477, 193.0137
3	Epigallocatechin 3,5,-di-O-gallate	5.95	609.0898	610.0958	[M-H]^−^	C_29_H_22_O_15_	3.848	259.0616, 215.0359, 193.0138
4	Dihydromyricetin isomer	6.10	319.0462	320.0532	[M-H]^−^	C_15_H_12_O_8_	4.345	257.0477, 193.0137
5	Hesperetin	7.32	607.0737	608.0802	[M-H]^−^	C_29_H_20_O_15_	3.103	259.0249
6	Myricetin-3′-O-β-d-xylopyranoside	7.37	449.0732	450.0798	[M-H]^−^	C_20_H_18_O_12_	3.780	NA
7	Myricetrin	7.72	463.0887	464.0955	[M-H]^−^	C_21_H_20_O_12_	3.472	287.0548, 151.0392
8	Quercetin-3-O-α-l-rhamnopyranoside	10.64	447.0963	448.1006	[M-H]^−^	C_21_H_20_O_11_	3.226	193.0135
9	Myricetin	11.72	317.0304	318.0376	[M-H]^−^	C_15_H_10_O_8_	3.905	191.0340
10	Quercetin	12.83	301.0357	302.0427	[M-H]^−^	C_15_H_10_O_7_	4.654	NA

NA: not assessed.

**Table 10:** Chemical constituents in ethanol extract of Vine.

No.	Compound	RT	Expected	Measured	Adduction	Formula	Delta	MS2 Ion
		(min)	(*m*/*z*)	(*m*/*z*)			(ppm)	(*m*/*z*)
1	Dihydromyricetin	4.34	289.0721	290.0790	[M-H]^−^	C_15_H_14_O_6_	5.104	203.0706, 125.0233
2	Epigallocatechin 3,5,-di-O-gallate	5.52	319.0460	320.0532	[M-H]^−^	C_15_H_11_O_8_	3.593	257.0477, 193.0137
3	Dihydromyricetin isomer	5.93	609.0895	610.0958	[M-H]^−^	C_29_H_22_O_15_	3.257	259.0616, 215.0359, 193.0138
4	Hesperetin	6.13	319.0461	320.0532	[M-H]^−^	C_15_H_11_O_8_	4.063	257.0477, 193.0137
5	Myricetin-3′-O-β-d-xylopyranoside	7.30	307.0737	608.0802	[M-H]^−^	C_20_H_20_O_15_	3.103	259.0249
6	Myricetrin	7.32	449.0730	450.0798	[M-H]^−^	C_20_H_18_O_12_	3.513	NA
7	Quercetin-3-O-α-l-rhamnopyranoside	7.71	463.0887	464.0955	[M-H]^−^	C_21_H_20_O_12_	3.407	287.0548, 151.0392
8	Myricetin	10.55	447.0936	448.1006	[M-H]^−^	C_21_H_20011_	3.159	193.0135
9	Quercetin	11.65	317.0304	318.0376	[M-H]^−^	C_15_H_10_O_8_	3.679	191.0340
10	Dihydromyricetin	12.73	401.0357	302.0427	[M-H]^−^	C_15_H_9_O_7_	4.654	NA

NA: not assessed.

## Data Availability

The original contributions presented in the study are included in the article, further inquiries can be directed to the corresponding author.
